# How to Address the Risk of HIV Transmission in Remission Studies With Treatment Interruption: The Low-Hanging Fruit Approach

**DOI:** 10.1093/infdis/jiz163

**Published:** 2019-07-02

**Authors:** Nir Eyal

**Affiliations:** Center for Population-Level Bioethics, Rutgers University, New Brunswick, New Jersey

**Keywords:** HIV, research ethics, analytic treatment interruption, HIV cure-related studies

## Abstract

Some HIV remission studies include a treatment interruption that seriously risks infecting participants’ sex partners with HIV. What, ethically, is owed to these nonparticipants? Until greater certainty emerges on what protections should be afforded nonparticipants of research studies, what I call a “low-hanging fruit” approach may help researchers and review bodies determine how to address infection risks to nonparticipants in these studies.

This supplement expounds a particular risk from the use of analytical treatment interruption (ATI) in some human immunodeficiency virus (HIV) cure-related trials: the risk of onward transmission to participants’ potential sexual partners (and *their* partners, and so forth). It pays special attention to remission studies with a “setpoint” ATI, in which the risk is elevated and less manageable (see the Introduction to this supplement [1]). How, ethically, should investigators and review bodies respond to these infection risks?

Writing on what is ethically mandated in this area is challenging in 2 ways. First, some debate persists on when studies that include ATIs are necessary in cure-related investigations for scientific reasons (see [2] in this supplement). The present perspective focuses on cure-related studies judged both important scientifically and impossible without an ATI, which thus represent real tensions between scientific and ethical needs.

A second challenge concerns so-called “moral uncertainty”. Let me explain. Bioethics writing on clinical trials that pose risk to nonparticipants (eg, risk of infection, radiation exposure, privacy breach, and stigma) gives few recommendations, and far fewer arguments, in their support [3–8]. Writers seldom specify, for example, whether nonparticipants are owed more protection or less protection than study participants. The resulting moral uncertainty (namely, uncertainty about basic moral recommendations, as opposed to one about facts) makes it hard to offer recommendations in this particular area of practice. Moral uncertainty is a central focus of contemporary ethics [9–11]. The current perspective tackles the moral uncertainty in our context procedurally. Throughout, its approach is that if a measure affords substantial protections to nonparticipants, it costs little time, effort, and money, and it involves no independent transgressions (eg, of participants’ privacy), then we can tentatively conclude that this measure is mandated for both ethical and regulatory purposes. That tentative recommendation may change once a fuller ethical theory makes more authoritative recommendations, based on fuller arguments. Let us call this procedural approach to addressing the risk of infection in HIV remission studies that include an ATI the “low-hanging fruit” approach.

The low-hanging fruit approach may sound obvious, but it parts ways with most contemporary philosophical approaches to moral uncertainty (again, uncertainty about basic moral recommendations, as opposed to uncertainty about facts). What most contemporary approaches recommend, when one is unsure what one should do, is to act in such a way that one maximizes the prospect that one does the right thing, or, alternatively, minimizes the prospect that one does wrong [9–11]. As an illustration, if the complex moral question whether you have the right to eat meat baffles you, better err on the side of moral precaution and avoid meat, because while eating meat is on some theories a terrible wrong akin to murder, avoiding meat is clearly within your right [10]. The low-hanging fruit approach adds that pragmatic considerations like cost minimization can legitimately have impact as well. If you are very unsure what is morally demanded, such pragmatic considerations may serve as tie-breakers or, depending on their weight, tilt the balance.

The low-hanging fruit approach also parts ways with the status quo in research ethics regulation and with current remission study practice. It demands some interventions that current regulation and practice reject, and rejects some that they espouse.

Risks of onward transmission can be tackled in 2 ways. Some measures reduce transmission risk. Others make any remaining risk ethically more justifiable (eg, by securing the consent of the party exposed to that risk). Many candidate measures of one kind or the other are conceivable. Let me evaluate candidate measures that strike me as distinctive to this area or as practically important to consider, discussing each at the stage of study conduct at which it occurs.

## RECRUITMENT

### Exclusion Criteria

Studies with an ATI regularly involve exclusion criteria that seek to protect study participants, including, for example, the participant’s history of CDC category C clinical events, history of cutaneous Kaposi sarcoma, and CD4 and nadir CD4 specifications (see eg, Jean-Daniel Lelièvre and Laurent Hocqueloux’s case earlier in this supplement, [12]). That protection of participants is important, but it does not always protect nonparticipants. To that aim, most relevant would be excluding candidate participants with high potential for infectiousness, for example, a record of a high setpoint (on the rare occasions these data are available), reported unsafe sex habits, and reported opposition to the added protections of nonparticipants recommended below. Only some of the latter demands are regularly in place.

For an example of a study that excludes potential participants on the basis of “significant risk of HIV transmission during treatment interruption in the opinion of the investigator, including evidence of unsafe sexual contacts,” see the Analytical Treatment Interruption in HIV Positive Patients (ISALA) study (NCT02590354). For examples of studies that exclude potential participants for refusing to comply with protective measures for nonparticipants, see the following studies: Tracking and Exploring the Source of Viral Rebound (NCT03117985); Towards HIV Functional Cure (ULTRASTOP; NCT01876862); Monitored Antiretroviral Pause in Chronic HIV-Infected Subjects With Long-Lasting Suppressed Viremia (APACHE; NCT03198325); and Biomarkers to Predict Time to Plasma HIV RNA Rebound (NCT03001128).

To reduce risk of onward transmission further, it can be justified to exclude candidate participants even based on their reports about the relevant characteristics of their sex partners in stable relationships. This is not part of the status quo, but, if and when these characteristics are reported, is easy to do. Of potential relevance here are reported physical or behavioral characteristics that would make a candidate participant’s partner(s) likelier to get infected. Seronegativity increases infection probability, but arguably not enough to make the absolute probability of secondary transmission high enough for exclusion when the partner is reported to be careful. Reported partner opposition to preexposure prophylaxis (PrEP) and to condom use usually indicates a probability high enough for exclusion. Also of relevance are reported physical or behavioral characteristics that would make a sexual partner be at high risk *from* getting infected, for example, chronic nonadherence in an unrelated condition, inasmuch as that indicates serious chance of antiretroviral therapy (ART) nonadherence. Reported multiplicity of partners would usually increase both the probability of and the risk from infection, both cumulatively and for each partner, for example, by making counseling of all on safe sex and on ART adherence more difficult.

Asking for a report about a sexual partner’s characteristics is not a violation of their privacy so long as they remain unidentified and without contact with the investigators, and the information remains confidential after all. The reporting is there to protect them. If the trialists are considering contacting partners to protect them, that presents a greater challenge, discussed below. There is no guarantee that the report is truthful but there is no major harm in using it to exclude participants who actually report partners at high risk of getting infected. Nor does this move settle all questions (eg, how many sexual partners is too many), but it provides the considerations in light of which to decide open questions.

### Informed Consent

During the informed consent process, discussing special precautions to prevent infection, as well as their limitations (eg, questions about the effectiveness of PrEP), is not only mandated for full disclosure; in our context it is also smart, as it helps select for participants likelier to accept and adhere to such exceptional operations, boosting the chances of safety and success. This can facilitate the exclusion of candidate participants at high risk of putting partners at risk, which should start during screening.

For other studies that place unidentified nonparticipants at risk, some ethicists have proposed community engagement, partly as a proxy for fuller informed consent, which is not feasible [6, 13, 14]; for our own context, input from people living with HIV was recommended on similar grounds [15]. However, a more relevant community here is future contacts at risk of getting infected who are yet unidentified. Interviewing representatives of that “community” on what remains a small risk for each of its members is a high-hanging fruit. Arguably it remains unnecessary.

### Payment

Some ethicists have recently argued that when studies are risky to individual participants, high payment is, in fairness, owed them [16, 17]. (Contrary to a popular conception, top ethical thinkers usually find high payment to study participants permissible) [18]. But high payment to participants cannot address risks to nonparticipants. So for our purpose, any case for high payment to study participants is largely moot.

## TRIAL OPERATIONS

### Education

To help preempt infection, frequent counsel on safe sex and contraceptive use is advisable throughout the trial. To give an analogy from a very different area, in the case of smoking cessation, brief counsel in every primary care physician visit is disliked by patients yet remains important [19].

### Restarting Preparedness

Frequent monitoring of viral load (potentially during in-home visits—a suggestion by Tim Henrich), with clear guidelines on when to restart ART, could prove crucial in eradication studies, where ART can be resumed immediately upon detection of virus [20]. However, in most remission studies, for the reasons stated in the introduction to this supplement, they are far from sufficient.

### Isolation

The difficulties in fully protecting sex partners, and especially partners in unstable relationships, through other channels may prompt the thought that geographical isolation should be used during ATIs in remission studies—conditioning participation on staying in the study facilities for the duration of the ATI. However, elsewhere in this supplement, my coauthor and I identify the problems with assigning isolation that role (see [21]).

### Moderately Limiting Placebo

Some cure-related studies assign participants to either an intervention arm or a placebo control arm. Participants in both arms then undergo an ATI. At HIV cure conferences, that practice was often controversial, with some doubting that it can ever be ethical to include a placebo arm in studies that involve an ATI. Recently, however, a consensus emerged that “If a placebo group is necessary for the findings of a study to be properly interpreted, it could be considered unethical not to include a placebo.” [22]

When one focuses strictly on participants, the case for this consensus is more straightforward than when sexual partners are taken into account. For participants, typically we cannot assume that placebo arm participation will pose greater net risk than participation in the intervention arm. On the contrary, intervention arms include both risks from the ATI and toxicity risks. While intervention arm participants do stand a chance at a cure, that chance is very small so missing it is not giving up much, from a medical standpoint. So if we should allow that participants in intervention arms can be treated ethically, for example, given their consent to take on risks and the social value of progressing toward a cure [23], we should likewise allow that participants in placebo arms can be treated ethically.

The same cannot be automatically said about sexual partners of participants in placebo arms. These sexual partners tend to face somewhat greater risks of getting infected than the sexual partners of participants in intervention arms (because in placebo arms, infectiousness is not mitigated by any chance at a cure). And their partners’ avoidance of toxicity has no impact on their health. Nor will these sexual partners typically have licensed the risk to their health. Sexual partners are not typically asked to consent to study risks. Nor were seronegative sexual partners of candidate participants represented in the consensus statement, as far as I know.

Still, perhaps we may argue that the social value of progressing toward a cure for HIV is high enough that when the validity of important cure studies requires a placebo arm, and when serious measures are taken to protect sexual partners, a placebo design is justified on balance.

## PROTECTIVE CARE FOR NONPARTICIPANTS AT RISK

Another host of potential protections of sexual partners from infection are ones that could be given directly to those partners—again in contrast to the status quo in research regulation in most areas of research. Like the measures discussed so far, interventions performed on partners range from the easy to the expensive and problematic.

Several issues are of ethical import and worth discussing here. First, any intervention on sexual partners requires contacting them. Is that a violation of their, or perhaps of study participants’, rights to privacy? This varies between partners in stable (and typically known) relationships and partners in unstable relationships.

When partners in stable relationships are reported not to know of the subject’s study participation or even her HIV status, hard questions may initially be thought to arise about confidentiality. Should the researchers report participation in studies that include an ATI to the partner (or to the local Department of Health—and should that department notify the partner)? However, in practice, this question should rarely arise. When researchers know in advance that a partner in stable relationships is expected to remain uninformed, this scenario usually calls for excluding the candidate participant in the first place. When, alternatively, what is reported is partners in unstable relationships, specific interventions on them, and even contacting them individually, usually become impracticable within constraints of budget and of their confidentiality. Either way, whether sexual partners are in stable or unstable relationships, participants’ medical confidentiality poses a lesser challenge than might initially seem.

### Protective Care

With the worry about confidentiality set to one side, let us ask about the protective intervention itself. Reported partners in stable relationships who are appraised of the subject’s HIV status and study participation can be easily offered counseling, for example, on safe sex and PrEP. Actually providing PrEP (eg, where it is not otherwise available) is more expensive but easily worth the cost, for investigators and sponsors alike [24].

More demanding is giving partners in stable relationships consent rights and specifically the right to refuse their partner’s participation, at least in advance of the trial. But similar consent rights were recommended by ethicists in a parallel context: “Where there are identifiable indirect participants and risks to them are significant, their informed consent should also be required for participation of the direct subject in research.” [6] The recommendation may be contentious in some studies that greatly benefit participants and society and place nonparticipants at small risk. But offering partners such “veto” rights in our context does *not* unfairly exclude candidate participants from any study that is vital for participants’ own safety; many of the cure-related studies involved are risky for participants as well [23]. Consent rights to partners are again very different from the status quo, but practicable in early phase trials, which require relatively few participants. So the procedural low-hanging fruit approach would support partner consent rights for the time being.

### Designating Partners in Stable Relationships as Study Participants

One possible measure would have been to designate partners in stable relationships as “study participants”, so as to offer them protections under CFR46 (an approach championed by some documents on participant rights in other kinds of studies) [25]. However, nonparticipants do not meet CFR46 criteria: information is not collected on them and they are not being studied. In plain English, too, there is a difference between being at risk from someone else being studied and actually being studied. When the partners are not being studied, they are not study participants. While instituting protective measures for them is important, and while one possible position is that those should coincide with protections that usually go to study participants only, these protections should be provided in a more appropriate way than pretending that anyone at risk is the subject of the study.

## MEASURES TO REDUCE HARM FROM INFECTION

### Treatment Support

There is virtually no way to completely stem the risk of secondary transmission in studies that include setpoint ATIs. Investigators need a plan for the event of suspected infection. If a potentially infected sexual partner can be contacted, investigators can help him or her in a variety of ways, for example, by testing for infection and facilitating access to HIV and other care as needed. Advance decisions and appropriate arrangements facilitate provision of services. That support is not part of the status quo either, but doable with advance planning, and potentially important.

One should remain clear about the rationale for helping infected nonparticipants, though. The rationale is not that they are owed as much by the law governing ethical research on human subjects (see my comment in, [26]). Part of it is the causal contribution of one’s actions to their infection. The upshot of those is complex because these actions are authorized and in the public interest and the infection would not materialize but for choices by the study participant and her sexual partner that a careful researcher will have tried to warn them against. But there is also a straightforward and important reason to provide this help to infected partners—the need to preserve public trust in the medical research enterprise and, specifically, in HIV cure-related research. When Jesse Gelsinger died in a (HIV-unrelated) gene therapy trial at the University of Pennsylvania in 1999, there were consequences not only for the study and research institution but for the field. The same might happen to HIV curative research. While sexual partners are not study participants, there is no guarantee that in the court of public opinion injury to a sexual partner would be judged any more leniently than injury to a participant.

### Limiting Conflicts of Interest

For similar reasons of protecting trust in research even in the event of an infection, a final recommendation is to rely on commercial sponsors only minimally. Following the Gelsinger event, investigators’ financial conflicts augmented suspicion of mixed motives, and the related distrust, which set back the entire field [27, 28]. In risky cure-related studies in general and in ones involving ATIs that place nonconsenting sex partners at risk in particular, commercial sponsorship is sometimes unavoidable, for access to drugs, reagents, and unpublished data, but should remain strictly minimal.

## SUMMARY

Both scientific and ethical uncertainties continue to shroud the ethics of ATI risk to nonparticipants in HIV remission studies. Until greater certainty emerges, the low-hanging fruit procedural approach makes recommendations across the different stages of study conduct. Those sometimes differ from and, in this writer’s view, should usually supersede the status quo in trial oversight and practice.

## Notes

Presented in part: Medical Study Risks to Nonparticipants: Ethical Considerations Workshop, Boston, Massachusetts, 11–12 June 2017.

## Notes

***Acknowledgments.*** The author gratefully acknowledges helpful comments from Steven Deeks and editorial assistance from Lisa Holtzman and Monica Magalhaes.

***Financial support.*** This supplement is sponsored by NIH: National Institute Allergy and Infectious Diseases [grant number 1 R01 AI114617-01A1 (HIV cure studies: risk, risk perception, and ethics)].

***Potential conflicts of interest.*** The author certifies no potential conflicts of interest. The author has submitted the ICMJE Form for Disclosure of Potential Conflicts of Interest. Conflicts that the editors consider relevant to the content of the manuscript have been disclosed.
